# Cross-Cultural Adaptation and Validation of a Surgical Neonatal Nursing Workload Tool for an Italian Context: The Italian Winnipeg Surgical Complex Assessment of Neonatal Nursing Needs Tool

**DOI:** 10.3390/nursrep15010018

**Published:** 2025-01-10

**Authors:** Emanuele Buccione, Floriana Pinto, Alessio Lo Cascio, Viola Palumbo, Kerry Hart, Allison Marchuk, Jessica-Lynn Walsh, Alexandra Howlett, The Italian Neonatal Nursing Workload Study Group, Laura Rasero, Davide Ausili, Stefano Bambi

**Affiliations:** 1 Department of Biomedicine and Prevention, University of Rome Tor Vergata, 00133 Rome, Italy; locascio.alessio@lamaddalenanet.it; 2 Neonatal Intensive Care Unit, Health Local Authority 3 Pescara, 65124 Pescara, Italy; viola.palumbo@asl.pe.it; 3 ASST Grande Ospedale Metropolitano Niguarda, 20162 Milan, Italy; floriana.pinto@ospedaleniguarda.it; 4 La Maddalena Cancer Center, 90146 Palermo, Italy; 5 Alberta Children’s Hospital, Alberta Health Services, Calgary, AB T3B 2X9, Canada; kerry.hart@albertahealthservices.ca; 6 Surgery Strategic Clinic Network, Alberta Health Services, Calgary, AB T3B 2X9, Canada; allison.marchuk@albertahealthservices.ca; 7 Cumming School of Medicine, University of Calgary, Calgary, AB T2N 1N4, Canada; jlawalsh@ucalgary.ca (J.-L.W.); alixe.howlett@albertahealthservices.ca (A.H.); 8 Department of Health Sciences, University of Florence, 50121 Florence, Italy; l.rasero@unifi.it (L.R.); stefano.bambi@unifi.it (S.B.); 9 Department of Medicine and Surgery, University of Milano-Bicocca, 20126 Monza, Italy; davide.ausili@unimib.it

**Keywords:** neonatal intensive care unit, care complexity index, newborns, preterm, nurses

## Abstract

**Background:** Complexity of care, adequate staffing levels, and workflow are key factors affecting nurses’ workloads. There remain notable gaps in the current evidence regarding clinical complexity classification and related staffing adjustment, limiting the capacity for optimal staffing practices. This study aimed to adapt and validate the Winnipeg Surgical Complex Assessment of Neonatal Nursing Needs Tool (WANNNT-SC) for an Italian context to allow the assessment of newborns admitted to NICUs. **Methods:** This was a validation study. **Results:** To evaluate the reliability of the tool among different professionals, a correlation test was performed using Pearson’s correlation, which revealed a strong correlation (r = 0.967, *p* = 0.01). In the test–retest phase, there was a significant correlation (r = 0.910 and *p* = 0.01). Using an analysis of variance, we found that the higher the I-WANNNT-SC score was, the higher the predicted death rate (F = 13.05 and *p* < 0.001). **Conclusions:** The Italian Winnipeg Surgical Complex Assessment of Neonatal Nursing Needs Tool represents the first tool available for an Italian context that aims to measure the nursing workload in neonatal intensive care. It could allow adjustments in nursing staffing based on NICU activities and patient needs. This study was prospectively approved by the local Ethics Committee “Palermo 1” (Protocol CI-NICU-00).

## 1. Introduction

Complexity of care, adequate staffing levels, and workflow are key factors affecting nurses’ workloads [1]. In practice, a nurse-to-patient ratio defined a priori does not reflect the severity of disease among admitted patients with different characteristics and needs [2]. There remain notable gaps in the current evidence regarding clinical complexity classification and related staffing adjustment, limiting the capacity for optimal staffing practices [3,4]. Further research on this topic, including a standard and valid measure of nursing workload, is urgently needed [5].

Nursing workload is defined as the ratio of demands or “task load” to available resources [6]. It encompasses physical, emotional, cognitive, and organizational aspects of work [6,7]. This has a significant impact on the quality of care, patient outcomes, and nurses’ well-being [8,9,10]. In neonatal and pediatric settings, nursing overtime (which can be a consequence of an increase in workloads) is associated with a higher frequency of health care–associated infections (HCAIs), an augmented rate of unplanned extubation, and an increased risk on bloodstream infections (BSIs) in very-low-birth-weight infants (VLBWIs) [11,12,13,14]. Furthermore, strategies to reduce HCAIs are based on the management of risk factors (e.g., line management, hand hygiene, and reduced use of antibiotics), and it has been reported that daily nursing overtime periods should also be considered as an important risk factor [11]. Evidence-based staffing strategies (monitoring nursing satisfaction, experience, and stress) can lead to improvements in patient safety and decreased rates of adverse outcomes [5].

A monocentric cross-sectional analytical study using the Nursing Activities Score (NAS) [15] to compare nursing workloads in the NICU, ICU, and CCU during morning and night shifts found that nurses in the NICU experienced significantly higher workloads than those in the ICU and CCU. These findings emphasize the importance of carefully considering nurse staffing distribution across NICUs [16].

In Italy, the number of admitted newborns, the size of the NICU, and the volume of activity in these units are highly variable [17]. Evidence also highlights an imbalance in terms of nurse/patient ratio (NPR) between units with low volumes of activity and those with high volumes; this finding implies higher nurse workloads in the largest NICUs [17]. A multicenter observational study in Italy involving 2769 pediatric nurses reported an average of 4.74 (SD ± 4.41) omitted activities per nurse in critical areas due to time constraints, including “informing and educating patients and/or family members” (omitted by 42.1%). The study revealed that 80% of nurses perceive care quality as less than excellent, and 50% rate patient safety below good. For the first time in pediatric care in Italy and Europe, key variables such as work environment, burnout, intent to leave, and omission of essential activities were analyzed, highlighting deviations from recommended staffing values and underscoring the need for policies ensuring safer pediatric care [18]. In this heterogeneous context, a tool that evaluates nursing workload based on the needs of newborns and their families is even more urgent.

The Winnipeg Surgical Complex Assessment of Neonatal Nursing Needs Tool (WANNNT-SC) is an instrument designed to assess the care complexity of newborns admitted to the NICU. It was selected because it is a modified version of the Winnipeg Assessment of Neonatal Nursing Needs Tool (WANNNT) [19], which is considered the most current instrument, reflecting the technological and clinical advancements that have occurred in neonatal intensive care over the past two decades. Furthermore, as described by Hart et al., the National Aeronautics and Space Administration Task Load Index (NASA TLX) [20], which is a recent tool used to measure the workload of neonatal nurses, has few surgery-specific indicators, and this metric does not quantitatively determine the staff required [21]. A previous study showed that a lower ratio of nursing care calculated using the WANNNT during the first seven days of admission was associated with an increased risk of mortality and morbidity in very preterm infants [22]. The WANNNT-SC, adding corrected gestational age and assigning an acuity score of 1.0 to very preterm infants in the first week of life, should not have this weakness. Furthermore, the Clinical Risk Index for Babies score (CRIB II), used to evaluate the criterion validity of the WANNNT-SC, was significantly better at predicting mortality than gestational age or birth weight alone. The time-dependent performance of the CRIB II was good throughout the first 90 days [23].

## 2. Materials and Methods

### 2.1. Study Design

This was a validation study to evaluate the reliability and validity of the Italian version of the WANNNT-SC [21]. The study was conducted following the COnsensus-based Standards for the selection of health Measurement INstruments (COSMIN) recommendations [24,25] in four phases from May 2023 to February 2024.

### 2.2. Translation, Back-Translation, and Cross-Cultural Adaptation

Authorization to use the tool was obtained from the authors. All WANNNT-SC items were first translated into Italian by a NICU critical care nurse who was a native speaker of Italian and certified as competent in the English language; then, a back-translation was performed by an expert NICU nurse who was a native English speaker and certified as competent in the Italian language [26]. The original WANNNT-SC tool and the translation obtained were compared by two researchers who confirmed its consistency and homogeneity. Cultural adaptation was needed; it was performed according to the Italian context and with the WANNNT-SC authors’ permission.

To adapt the WANNNT-SC tool to an Italian context, several items were modified [27].

The “THAM infusion” item was replaced by “Sodium Bicarbonate infusion”. Sodium bicarbonate and Tris-hydroxymethyl-aminomethane (THAM) have both been used to correct metabolic acidosis in neonates [28]. Sodium bicarbonate is commonly used in Italy.

The Neonatal Emergency Transport Service (NETS) is a specialized medical service that provides emergency transport for critically ill or premature newborns. This service is typically offered by hospitals or health systems equipped with NICUs and aims to safely transport newborns who require immediate medical attention, specialized care, or transfer to a NICU that can better meet their needs. In Canada, the health system is managed at a provincial level; therefore, less acute infants are frequently transferred from high-risk centers (classified as Level III) to low-risk regional hospitals (classified as Level II) to create space for more acute patients. However, in Italy, regional governments have autonomy to legislate issues related to healthcare, resulting in regional organizational variations in resources and models [29]. Approximately 30% of low-level delivery hospitals that handle fewer than 500 births/year are still active, and there are more level II NICU beds than are needed, while level III–IV beds are inadequate, forcing return transport as soon as the clinical conditions of the newborn allow it [30]. Therefore, the authors decided to modify the items based on the type of transport. For these reasons, the “Transfer out of town” and “transfer to another inner-city hospital” items were replaced by “Neonatal Emergency Transport Service (NETS)” and “Back transport”, respectively.

### 2.3. Face Validity

The face validity phase involved nine experts with extensive clinical experience in the NICU. The panel included three head NICU nurses (33.33%), four NICU nurses (44.44%), and two cardiac NICU nurses (22.22%). Panelists provided feedback on the instrument’s face validity, specifically its clarity and adequacy in measuring neonatal nursing workload. Between 1 June and 30 June 2023, each expert completed an online questionnaire, with the option to provide suggestions if necessary [25]. Participants also attended an educational training session on the tool. Anonymity was maintained throughout the process, and experts from diverse nursing contexts and clinical settings were involved to ensure the phase’s precision and comprehensiveness.

### 2.4. Internal Validity

Inter-rater reliability was tested from the 1–31 July 2023 using a double-blind process to evaluate the constancy of the measure among different users over time [25]. After an educational course focused on the tool and its use, two nurses performed their evaluations at the same time through in-person evaluations. The test–retest reliability phase was performed from the 1–31 August 2023. The time interval was defined a priori using the duration of the entire shift (approximately 8 h). Evaluations were performed in person at the start of the shift and by another nurse at the end. In light of the objective set, only the clinical variations in the newborns evaluated at the beginning of the shift were considered. To avoid selection bias, all emergency activities (NETS, calls from the delivery room, and calls from the surgery room) and new admissions were included.

### 2.5. External Validity

From 15 November 2023 to 15 February 2024, a test of criterion validity was performed to evaluate the degree of agreement between the tools and a gold standard [25]. The gold standard measure chosen was the risk of mortality, calculated using the CRIB II to highlight the correlation between care complexity and risk of mortality [31]. A recent study found the CRIB II score to have good predictive performance for overall mortality in VLBWIs, and it was highlighted as a proper risk adjustment tool for quality improvement initiatives to reduce mortality [23].

### 2.6. Tools

The WANNNT-SC is a nursing workload tool used in surgical and complex-medical-needs NICUs [21]. It is a modified version of the WANNNT [19]. The WANNNT was developed using an acuity–quality approach. A group of experts in neonatal nursing identified several specific indicators to classify lower- to higher-level nursing workloads. Fractions were used rather than nurse/patient ratios to create more manageable combinations of nursing assignments. For example, a “1.0” value means that one nurse was required to care for the newborn for the entire shift [19]. The WANNNT-SC includes several modifications. The authors added the corrected gestational age, divided the tool into systems, and included indicators related to neurocritical and surgical care [21]. Neurodevelopmental care, such as transfers for skin-to-skin care, and certain subjective aspects of workload, such as interacting with parents, were not explicitly addressed but, as an inherent patient care activity, were expected to be captured by corrected gestational age scoring. Indicators, including preoperative and postoperative care and transfers out of the unit or to other sites, were added. The WANNNT-SC considered only direct bedside care, excluding admitting nursing staff and charge nurses. The WANNNT-SC is clinically valid and reliable. This tool accurately captures and standardizes perceived work intensity from both charge nurses and senior nursing staff, indicating inter-rater reliability. Indeed, we found that the inter-rater kappa was 0.73 (CI 0.60–0.87). No significance was found when comparing the mean difference between charge nurse and senior nurse use of the WANNNT-SC (*p* = 0.94, SD 0.32, CI −0.077–0.083) [21].

The Clinical Risk Index for Babies (CRIB) II score is a tool used to assess the risk of mortality in newborns, particularly those in NICUs. It is an updated version of the original CRIB score, designed to predict the likelihood of death in preterm and low-birth-weight infants based on clinical variables [32]. Parry et al. developed the CRIB II for infants up to 1 h after admission to neonatal intensive care. Authors used the Akaike information criteria to identify the best model, which included birth weight, gestational age, gender, body temperature at admission, and base excess. An increased CRIB II score is related to an increased mortality risk. The possible CRIB II scores range from 0 to 27 [31].

### 2.7. Sample and Setting

**Figure 1:**
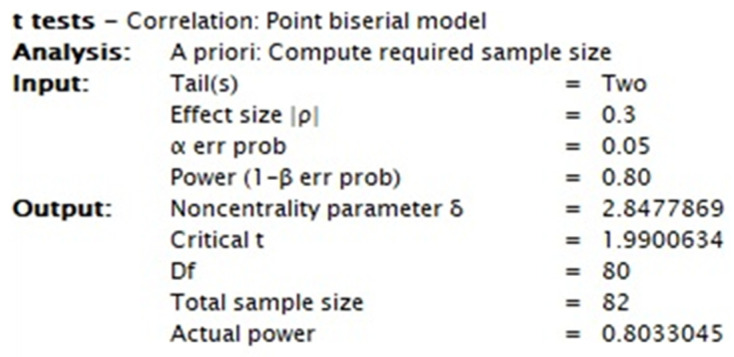
Protocol of power analysis.

This study enrolled neonatal patients admitted to the NICU with diagnoses related to medical, surgical, neurological, and cardiovascular diseases. Upon admission to the NICU, each newborn was evaluated using the Italian versions of the WANNNT-SC and the CRIB II. A reliable and suitable sample size of 82 evaluations was determined using an a priori power analysis for the criterion validity analysis, as shown in Figure 1.

### 2.8. Ethical Considerations

The study protocol was approved by the Local Ethics Committee “Palermo 1” (Protocol CI-NICU-00). The nurses and infants who participated in this study did not receive any intervention. This research was carried out according to the principles of the original Declaration of Helsinki and its subsequent amendments. Written informed consent was obtained from the parents or legal guardians of the neonates included in the study upon admission to the Neonatal Intensive Care Unit. Participation was voluntary and anonymous. The data were stored and managed according to the current Italian personal data protection code (Legislative Decree no. 196 of 30 June 2003). Data were collected and analyzed anonymously.

### 2.9. Statistical Analysis

The dataset was stored in the ZENODO consultable using the identifier 10.5281/zenodo.13823401. Descriptive statistics were reported as appropriate after testing continuous variables for normality using the Shapiro–Wilk test. The frequency and percentage were reported for nominal variables, whereas the median and interquartile range [IQR] or mean and standard deviation (SD) were calculated for continuous variables with normal distributions. The Pearson coefficient was used to evaluate correlations between the inter-rater and test–retest phases and between scores obtained with the tools during the external validity phase. Finally, an analysis of variance was performed to estimate the magnitude of the association between the predicted mortality risk and scores on the Italian version of the WANNNT-SC. The threshold for statistical significance was set at a *p* value of less than 0.05. Statistical analysis was performed using IBM SPSS Statistics for Windows, Version 22.0. Armonk, NY, USA: IBM Corp., whereas statistical power analysis was performed using G*Power 3.1. [33].

## 3. Results

### 3.1. Face Validity

The panelists had a median of 12 years [9,10,11,12,13,14,15] of experience in NICU or cardiac NICU settings. They represented children’s and general hospitals across northern, central, and southern Italy. The overall agreement on face validity was 92.3%, as shown in Table 1.

### 3.2. Inter-Rater Reliability

The ratings were given by 30 nurses, of whom 18 (60%) were female. The median experience of the evaluators in the NICU was 5 [4,5,6,7,8,9,10] years. A total of 239 pairs of double-blind evaluations were performed. To evaluate the reliability of the tool among different professionals, a correlation test was performed using Pearson’s correlation; the test revealed a strong correlation (r = 0.967, *p* = 0.01).

### 3.3. Test–Retest Reliability

This phase aimed to assess the instrument’s reliability over time, defined a priori as a nursing shift (8 h). A total of 286 paired evaluations were conducted by the same nurse, and Pearson’s correlation was applied. A significant correlation was observed, with r = 0.910 and *p* = 0.01.

### 3.4. Criterion Validity

**Figure 2:**
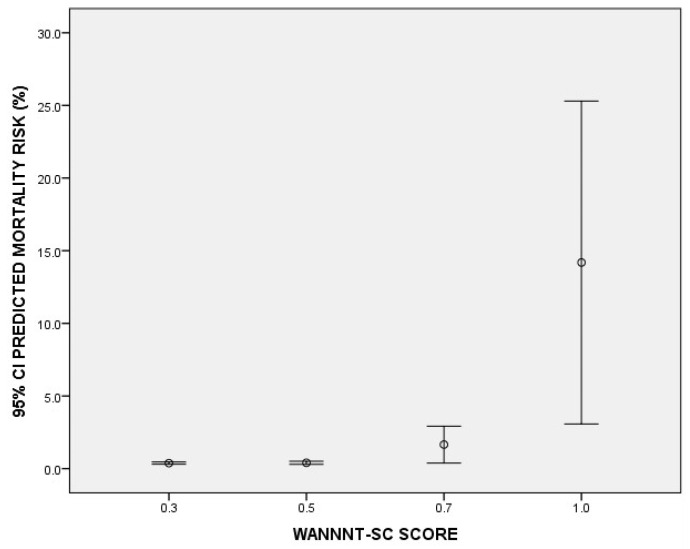
Mean predicted mortality risk (CRIB II score) according to WANNNT-SC score.

Lastly, this phase aimed to assess how well the tool correlates with an established measure. A total of 100 newborns were included in this study. The median weight and gestational age were 2540 [1950–2850] grams and 37 [34,35,36,37,38] weeks, respectively. A large number of newborns were reported to have low/medium care complexity: 34% (*n* = 34) and 32% (*n* = 32) were reported to have scores of 0.3 and 0.5, respectively. Nineteen percent of newborns (*n* = 19) needed intensive care with a score of 0.7, and only 15% (*n* = 15) needed a one-to-one nurse-to-patient ratio with a score of 1. In order to compare the expected mean mortality rates between the different complexity clusters, an analysis of variance was used. We found that the higher the WANNNT-SC score was, the higher the predicted death rate (F = 13.05 and *p* < 0.001). In fact, the groups “0.3”, “0.6”, “0.7” and “1” were reported to have average mortality risks of 0.37% (95% IC: 0.30–0.45), 0.40% (95% IC: 0.29–0.50), 1.65% (95% IC: 0.38–2.91) and 14.18 (95% IC: 3.07–25.29), respectively (Figure 2).

## 4. Discussion

This study aimed to adapt and validate the WANNNT-SC tool in an Italian context. Adaptation was necessary to account for differences in the healthcare systems of the two countries, as well as variations in nursing roles, education, and practices. These differences are evident in daily clinical practice, influencing nursing staffing standards and the activities performed by nurses [34]. We found strong inter-rater reliability and good stability over time. Furthermore, the Italian version of the WANNNT-SC presented a strong correlation with CRIB II, where mortality risk increased as the acuity score increased. This is important because, before the WANNNT-SC, no ad hoc tools had been developed or validated from other contexts for the assessment of care complexity in neonatal care settings in Italy; this made a correlation between nursing workload and nursing-sensitive outcomes impossible. Using the Italian version of the WANNNT-SC, it will be possible to describe a direct association between increased nursing overload and higher rates of HCAIs, unplanned extubations, bloodstream infections, and missed care [10,11,12,13,14] in an Italian context. There is reportedly a significant interest in validating acuity scores to ensure proper patient assignments. Instead of relying on fixed ratios, often determined by law, nurses aim to assess patient acuity and assign appropriate ratios based on the individual needs of the patients [35]. The daily use of the WANNNT-SC could lead hospital managers to adjust nursing staffing based on neonatal intensive care activities and could help optimize the assignment of nurses according to patient needs. NICUs can have tremendous variability in patient acuity and a wide range of possible admission diagnoses, requiring many different types of nursing skills [17,36], and a defined nurse/patient ratio does not reflect the severity of the disease among admitted patients [2]. Furthermore, the Italian organizational standard for perinatal care [37] defines a nurse-to-patient ratio of 1:2 in NICUs without a specific description of the clinical care needs, thus increasing risk of over- or underestimation of the necessary number of nurses. Lastly, the demanding and intricate nature of the workload in NICUs is a key factor contributing to the stress levels of NICU nurses [38]. Better workload management could improve job satisfaction in this area.

Another important aspect that the WANNNT-SC considers is the relational and educational activities focused on the parents and families of the newborn—activities that are often not considered in the evaluation of Italian nurses’ workload in the NICU. Lastly, as mentioned earlier, due to organizational, educational, and practical differences reflected in the daily activities of nurses in NICUs, we believe the results may not be generalizable to other countries. However, the instrument has demonstrated a solid structure that can be adapted to the specific context through a process similar to the one used in this study.

### Limitations

This study had some limitations. During the evaluation of criterion validity, only mortality risk was assessed, but future studies will be necessary to relate the Italian version of the WANNNT-SC to other nursing-sensitive outcomes (pressure injuries, HCAIs, and drug administration errors). Furthermore, this study focused only on patient acuity. Other factors also affect the nursing workload, such as environmental aspects (the layout of the NICU), organizational aspects, and the nursing skill mix. Unfortunately, as described, in Italy, there is high variability in organizational characteristics and work environments among NICUs as well as an uneven distribution of human resources in relation to the volume of activity [17], with a critical shortage of nurses in pediatric settings [39]. However, precisely defining each patient’s needs using the WANNNT-SC could be a good starting point.

## 5. Conclusions

The Italian Winnipeg Surgical Complex Assessment of Neonatal Nursing Needs Tool represents the first tool available for an Italian context that aims to measure the nursing workload in neonatal intensive care. It showed strong validity, reliability, and good correlation with the CRIB II score. The I-WANNNT-SC could allow adjustments in nursing staffing based on NICU activities and patient needs. Future studies are necessary to allow a description of the Italian context, to compare NICUs of different levels, and to objectively relate nursing outcomes to their workload and stratify the risks accordingly.

## Figures and Tables

**Table 1:** Evaluations by expert panel.

Expert	Assessed Dimensions									
	Appropriateness of Syntax	Correct Spelling of Words	Correct Sentence Structure	Appropriateness of Font Size and Spaces	Readable Instrument	Clarity and Unambiguity of Items	Adequacy of Instructions	Construction and Format Are Well Thought Out	Appropriateness of the Difficulty Level	Reasonableness of the Items in Relation to the Objective
1	Y	Y	Y	Y	Y	Y	Y	Y	Y	Y
2	Y	Y	Y	N	N	N	Y	Y	Y	Y
3	Y	Y	Y	Y	Y	Y	Y	Y	Y	Y
4	Y	Y	Y	Y	Y	Y	Y	Y	Y	Y
5	Y	Y	Y	Y	Y	Y	Y	Y	Y	Y
6	Y	Y	Y	Y	Y	Y	Y	N	Y	Y
7	Y	Y	Y	Y	Y	Y	Y	Y	Y	Y
8.	Y	Y	Y	Y	Y	Y	N	Y	Y	Y
9	Y	Y	Y	Y	Y	N	N	Y	Y	Y
% agreement	100	100	100	89	89	78	78	89	100	100
% OVERALL	92.3									

Legend: Y, yes; N, no.

## Data Availability

The dataset used in the current study is available in ZENODO and consultable using the identifier 10.5281/zenodo.13823401.

## Public Involvement Statement

There was no public involvement in any aspect of this research.

## Guidelines and Standards Statement

This manuscript was drafted against the COnsensus-based Standards for the selection of health Measurement INstruments (COSMIN). A complete list of reporting guidelines can be accessed via the Equator Network: https://www.equator-network.org/.
